# Emerging Technologies With Potential Care and Support Applications for Older People: Review of Gray Literature

**DOI:** 10.2196/17286

**Published:** 2020-08-11

**Authors:** Sarah Abdi, Luc de Witte, Mark Hawley

**Affiliations:** 1 Centre for Assistive Technology and Connected Healthcare School of Health and Related Research The University of Sheffield Sheffield United Kingdom

**Keywords:** artificial intelligence, internet of things, mobile phone, robotics, emerging technologies, older people, care and support

## Abstract

**Background:**

The number of older people with unmet care and support needs is increasing substantially due to the challenges facing the formal and informal care systems. Emerging technological developments have the potential to address some of the care and support challenges of older people. However, limited work has been done to identify emerging technological developments with the potential to meet the care and support needs of the aging population.

**Objective:**

This review aimed to gain an overview of emerging technologies with potential care and support applications for older people, particularly for those living at home.

**Methods:**

A scoping gray literature review was carried out by using the databases of 13 key organizations, hand searching reference lists of included documents, using funding data, and consulting technology experts. A narrative synthesis approach was used to analyze and summarize the findings of the literature review.

**Results:**

A total of 39 documents were included in the final analysis. From the analysis, 8 emerging technologies were identified that could potentially be used to meet older people’s needs in various care and support domains. These emerging technologies were (1) assistive autonomous robots; (2) self-driving vehicles; (3) artificial intelligence–enabled health smart apps and wearables; (4) new drug release mechanisms; (5) portable diagnostics; (6) voice-activated devices; (7) virtual, augmented, and mixed reality; and (8) intelligent homes. These emerging technologies were at different levels of development, with some being trialed for care applications, whereas others being in the early phases of development. However, only a few documents mentioned including older people during the process of designing and developing these technologies.

**Conclusions:**

This review has identified key emerging technologies with the potential to contribute to the support and care needs of older people. However, to increase the adoption of these technologies by older people, there is a need to involve them and other stakeholders, such as formal and informal carers, in the process of designing and developing these technologies.

## Introduction

### Background

Many older people are likely to require care and support in their later lives due to living with limiting long-term conditions [1,2]. These include support with activities related to mobility, daily living, and social life [2]. In the United Kingdom, for instance, it is estimated that around 20% of men and 30% of women aged 65 years and above currently require care and support with at least one activity of daily living (ADL), such as bathing and dressing [3]. In addition, many older people who require care and support prefer to continue living in their own homes, which is also a priority to several local authorities in the United Kingdom [4]. However, many of those are left with unmet needs due to the challenges facing the formal and informal care systems in the United Kingdom, such as limited funding to health and social care and physical and mental burden on family carers [5-7]. For example, a recent analysis of data from wave 7 of the English Longitudinal Study of Aging revealed that 55% of older people who have difficulty with at least one ADL received no formal or informal support [6]. Addressing the care and support needs of an aging population has, therefore, become an urgent health and social care priority, given the negative impact unmet needs have on older people as well as on the care systems [5,6].

There is a growing body of evidence demonstrating the potential of technology to meet older people’s care and support challenges. For example, data from recent systematic reviews have demonstrated the positive impact of a number of technologies on the physical and mental health of older people as well as on the social aspects of their lives [8-12]. Recent reports have also shown that older people enjoy the experience of using technology, are willing to engage with technology-based interventions, and tend to acknowledge its importance as a means to facilitate daily activities and communication [13-15]. However, older people adopt technology at lower rates compared with other age groups [16,17]. For example, more than 60% of internet nonusers in the United Kingdom are adults aged 75 or above [17]. Some of the main barriers that could influence and predict technology adoption by older people include lack of confidence in digital skills and lack of understanding of perceived value and positive impact of technology on their quality of life [13,14,16,18-22]. Many older people also face physical changes, such as cognitive decline, memory problems, and motor and sensory changes, that limit their use of available technologies [16,21,22]. A possible way to facilitate successful implementation of technologies targeting the care and support challenges of older people is to address the barriers to adoption during the process of technology design and development [23-26]. To achieve this, it will be important first to gain an overview of early phase technologies with potential care and support applications for older people. These technologies are increasingly referred to as emerging technologies [27].

Emerging technologies are early stage technological developments with high potentials that are yet to be demonstrated [27]. In recent years, the use of the term has encountered significant growth, paralleled with efforts to define what characterizes an emerging technology [28]. One of the main characteristics of emerging technologies commonly mentioned in the literature is their ability to provide investing bodies a change in *status quo* by exerting economic or social impact [27,29-31]. In addition, two other characteristics that have general agreement among academic scholars are growth or increase over time and novelty or newness [28,32,33]. These attributes were acknowledged in a notable definition of emerging technology in the literature by Rotolo et al [28]. Rotolo et al [28] analyzed 12 definitions from the social science domain and identified 5 key attributes of an emerging technology: (1) radically novel, (2) relatively fast growing, (3) coherence persisting over time, (4) potential to have socioeconomic impact, and (5) uncertainty and ambiguity about potential applications [25]. However, despite these efforts, the challenges of defining and operationalizing the detection of emerging technologies are well acknowledged in the literature [27,28,32,33]. For example, using traditional quantitative measures, such as patent analysis, to examine the potential socioeconomic impact is acknowledged to be challenging [28]. Similarly, operationalizing all key attributes of emerging technologies at the same time is considered to be difficult, given that the available data sources can carry different pieces of information [32]. Overall, it is acknowledged that the concept of emerging technology and methods of operationalizing the term is still evolving [27,28,32,33]. Therefore, methods to identify emerging technologies will depend on the study objectives and information and data sources. For the purpose of this review, the term *emerging technologies* has been operationalized as *technological developments that are novel and rapidly growing and have a potential socioeconomic impact*.

Some emerging technologies may help overcome common barriers of engagement with technology for older people. For example, recent advances in artificial intelligence (AI)–based conversational platforms are said to simplify end users’ engagement with digital technologies by reducing the need for complex skills to navigate websites or other interfaces [34,35]. Arguably, this could help address older people’s limited digital skills. Similarly, self-driving vehicles have seen significant advances recently and could soon help address mobility needs of older people [36]. However, despite these potential benefits, there is limited evidence synthesis that focuses on identifying emerging technologies with potential care and support applications for older people. Most of the recent works have focused on exploring the effectiveness and perceptions of specific technologies among older people [12,14,25,37-40]. In one of the few recent reviews on this topic, Sapci and Sapci [41] investigated current research evidence on elderly care technology, in particular novel remote monitoring technologies [41]. They reported an increased interest in recent years on exploring the potential of sensor-based smart homes, robotic technologies, and AI to support elderly care. They also highlighted that the latter would play an increasing role in remote monitoring technologies. However, their review focused mainly on monitoring technologies. Arguably, there is a need to gain an overview of recent technological developments, given that other technologies might play a role in elderly care in the future. An overview of emerging technologies could also help identify developments that might not be currently used to meet the care needs of older people but could potentially meet their needs in the future.

Therefore, a scoping review was conducted to gain an overview of emerging technologies with potential care and support applications for older people, particularly for those living at home. Literature searching was restricted to gray literature documents. This is because most of the overviews and analyses around emerging technologies tend to be found in the gray literature documents, such as funding bodies and science and technology institutes’ reports [42-44]. For example, the World Economic Forum and the Massachusetts Institute of Technology, renowned institutes in science, business, and technology, publish regular reports on emerging technologies [35,36]. In addition, most of these reports focus on emerging technologies with potential social or economic impact, which could provide insights into an attribute that is difficult to operationalize in empirical literature [28]. Analyzing these reports could also provide timely information about emerging areas of technological developments, given the quick nature of publishing in gray literature as opposed to research literature [45-47].

## Methods

### Study Design

A scoping review design based on the Arksey and O’Malley original and enhanced framework was used to conduct this review [48,49]. A scoping review design was deemed appropriate as this method allows to search the literature systematically and summarize and disseminate the findings of the literature search [48,49]. Following a systematic approach in searching gray literature documents was important to improve the reproducibility of the review and overcome some of the challenges encountered when conducting gray literature searches, such as lack of standard indexing and nontraditional formats of documents [50,51]. The Arksey and O’Malley original and enhanced framework recommends 6 steps in conducting a scoping review: (1) identifying the research question; (2) identifying relevant documents; (3) selecting the documents; (4) charting the data; (5) organizing, summarizing, and reporting the findings; and (6) consulting stakeholders (optional). The following sections describe the methods used to conduct the first 5 steps.

### Identifying the Research Question

This review aimed at answering the following research question: *What is known from the existing gray literature about emerging technological developments that could have potential care and support applications for older people living at home?*

### Identifying Relevant Documents

Identifying information sources for gray literature review depends largely on the objective of gray literature search [45,46,50]. For this review, reports from key organizations and data on ongoing research were deemed suitable to gain an overview of emerging technologies with potential care and support applications for older people. Several strategies have been used to identify relevant documents and minimize potential bias resulting from using a single search strategy for gray literature reviews [45,50]. First, key organizations were identified by running a Google search and based on their potential to publish documents related to the investigated topic. The web pages of these organizations were then searched for relevant documents using publication databases or free text search engines. Table 1 summarizes the strategies used to search each database.

In addition, reference lists of included documents were hand-searched to identify more relevant documents. Technology experts were also consulted to identify organizations and key publications on the topic of emerging technology. Funding data were used as a complementary resource to understand ongoing research activities and provide timely information about technology developments [28]. Data from the Engineering and Physical Sciences Research Council (EPSRC) were identified for this purpose. EPSRC was selected as it is the main funding body for engineering and physical sciences research in the United Kingdom [52]. Data identified included research projects currently funded in relevant research areas, including engineering, information and communication technologies, health care technologies, AI, robotics, human-computer interaction, pervasive and ubiquitous computing and sensor and instrumentations, assistive technology, rehabilitation, and musculoskeletal biomechanics. The funding amount of the key research areas and EPSRC experts’ magazine (*Pioneer*) were also analyzed for the last 5 years (2015-2019).

**Table 1:** Search strategy conducted on websites of key organizations.

Organizations	Search methods	Search terms
Deloitte	The website search engine was used to look for publications. The date was limited to after January 2015.	“Emerging” or “new” technologies were used as search terms.
Department of Health and Social Care	The website search engine was used to look for publications. The date was limited to after January 2015.	“Emerging” or “new” technologies were used as search terms.
NHS^a^	The website search engine was used to look for publications. Date was limited to after January 2015.	“Emerging” or “new” technologies were used as search terms.
Nuffield Trust	Search was limited to “Health and Social Care finances and reform,” “Older people and complex care,” and “New models of Healthcare delivery.”	“Emerging” or “new” technologies were used as search terms.
MIT^b^	All issues of MIT’s Technology review were screened from January 2015.	N/A^c^
World Economic Forum	All reports under the publication and white paper sections were screened from January 2015.	N/A
King’s Fund	All publications under the publication section were screened. No restriction was made on the topic. The date was limited from January 2015.	N/A
Nesta	All publications in the health, AI, data analytics, and future scoping sections were screened. The date was limited from January 2015.	N/A
Gartner	All reports in the special reports section were screened. In addition, an advanced search was conducted using the website search engine using “Hype cycles” and “Knowledge and Innovation” filters.	N/A
European Parliament and Commission	All publications, research, and reports under the EU^d^ publications section were screened from January 2015.	N/A
Royal Society	All reports under the publications section were screened from January 2015.	N/A
United Nations	All publications in the multimedia library of the Economic and Social Affairs were screened. The date was limited from January 2015.	N/A
World Health Organization	List of publications in Health Technologies was screened from January 2015.	N/A

^a^NHS: National Health Service.

^b^MIT: Massachusetts Institute of Technology.

^c^N/A: not applicable.

^d^EU: European Union.

### Selecting the Documents

Documents were selected as per predefined inclusion and exclusion criteria. In brief, documents were selected if they described an emerging technological development that could potentially be used to meet the care and support needs of older people living in their own homes. This review focused on the following care and support domains: mobility, self-care and domestic life, social life and relationships, psychological support, and access to health care. These domains were identified in recent research as important areas of care and support for older people living at home [2]. It is important to also note that the technology did not necessarily need to be developed for older people. This is because emerging technologies are in early development stages, and some ambiguity might still be associated with their potential users [28]. Textbox 1 summarizes the inclusion and exclusion criteria.

Documents were screened in 3 steps: (1) screening the headings or titles of the documents; (2) screening the summaries of the documents such as executive summaries, overviews, and key findings; and (3) screening full text of the documents. The screening process was conducted primarily by the first author (SA). An opinion from a second reviewer (LW or MH) was sought in case of uncertainty. A PRISMA (Preferred Reporting Items for Systematic Reviews and Meta-Analyses) flowchart was used to summarize the screening and selection process.

**The inclusion and exclusion criteria used to select documents.**

Documents were included if they

- Described an emerging technology and used the term “emerging” explicitly or mentioned attributes of an emerging technology such as fast, new, novel, rapid, or potential societal or economic impact;
- Described a potential application of the emerging technology in one of the following care and support domains: mobility, self-care and domestic life, social life and relationships, psychological support, and access to health care;
- Described technological development that could potentially be used to support older people in their own homes or within a community setting;
- Were reports or reviews published by the identified key organizations;
- Publication date between January 2015 and July 2019;
- Were published in English

Documents were excluded if they

- Described technical developments without mentioning potential applications;
- Described non care and support applications (eg, water/food security, business, and marketing);
- Focused exclusively on technologies used in a clinical setting (eg, during surgery or in hospitals). However, technologies used in clinical practice with potential uses in home such as remote monitoring were included;
- Were blogs or news articles;
- Were documents with highly restrictive use policy

### Charting the Data

Data were extracted from documents deemed eligible for the final analysis using a data extraction form on Microsoft Excel. The form included the following information: the name of the organization, the year and title of the publication, the purpose of the document, methods used or sources of evidence, description of the technology, and potential care and support applications for technological development.

### Organizing, Summarizing, and Reporting the Findings

The data extracted were summarized using a narrative synthesis approach [53]. The analysis started with the development of an initial description of the key findings of the included documents. To facilitate this step, a summary table was developed listing the main emerging technologies and application areas mentioned in each document. The next phase aimed to identify the categories of emerging technologies with potential care and support applications. It involved comparing and contrasting findings within each document as well as across the full data set. This phase of analysis revealed the complexity of the topic investigated. For example, some technologies were identified as emerging breakthroughs in some documents, whereas in other documents, these were identified as use cases or application areas of other technologies. This necessitated the distinction between emerging technologies that have enabled recent technological advances (enabling technologies) and those that could be used to meet the care and support needs of older people. This review focuses on reporting the findings of the latter. A description of each of these technologies was developed. Some examples were also provided on how these technologies were enabled by the emerging enabling technologies.

## Results

### Summary of Literature Search

**Figure 1:**
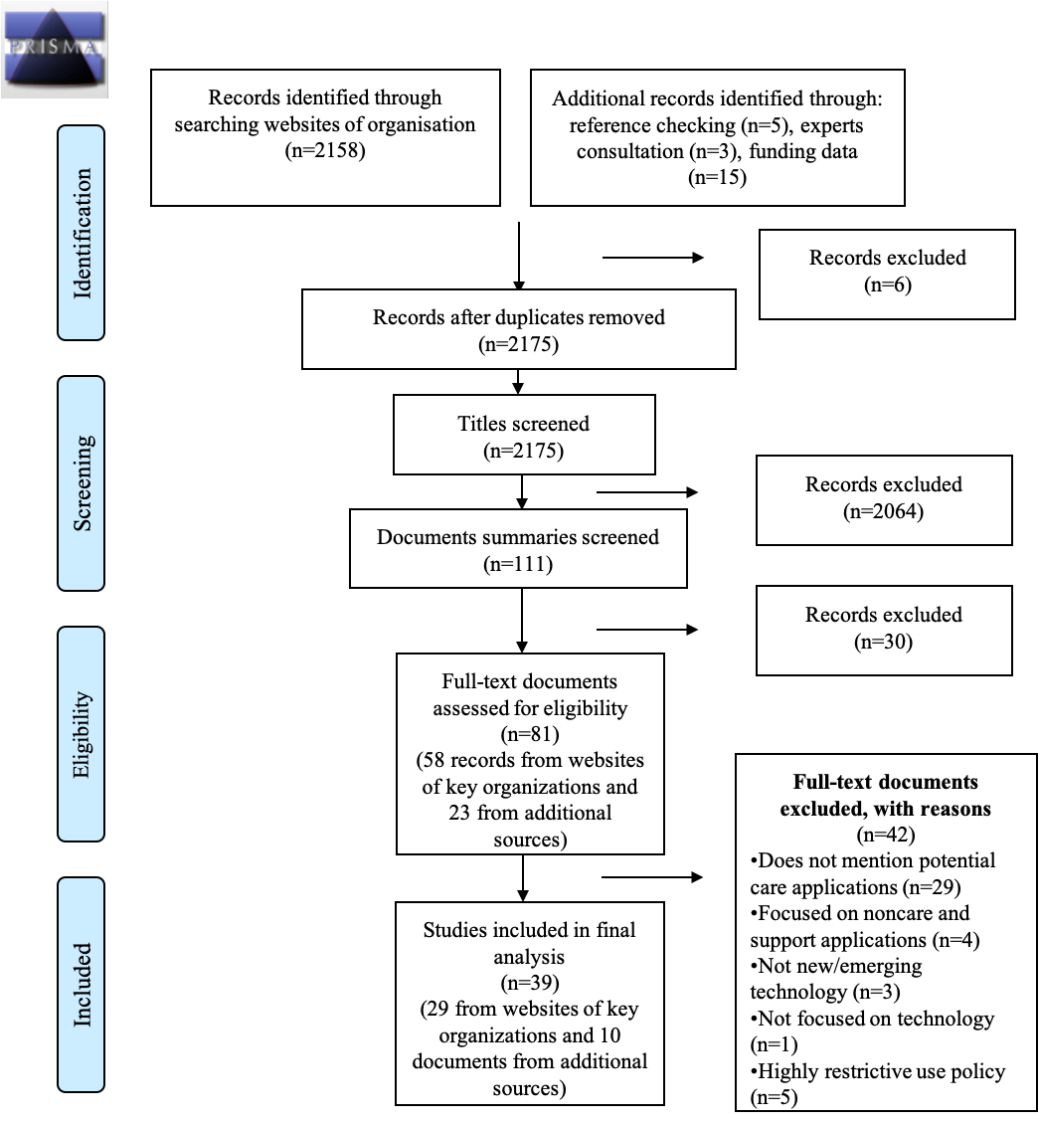
A summary of the screening and selection process using a PRISMA (Preferred Reporting Items for Systematic Reviews and Meta-Analyses) flowchart.

A total of 2158 records were screened from organizations’ websites, of which 58 were found eligible and were included in the full-text assessment phase. In addition, 23 records were identified from other sources and were screened for eligibility (5 from hand searching-included documents, 3 from speaking to experts, and 15 from funding data). A total of 39 documents were included in the final analysis. Figure 1 summarizes the screening and selection process using a PRISMA flowchart. The characteristics of the documents included in the final analysis can be found in Multimedia Appendix 1 [54-92].

### Key Findings

The analysis identified the following 8 emerging technologies that could have potential care and support applications for older people: (1) assistive autonomous robots; (2) self-driving vehicles; (3) AI-enabled health smart apps and wearables; (4) new drug release mechanisms; (5) portable diagnostics; (6) voice-activated devices; (7) virtual reality (VR), augmented reality (AR), and mixed reality (MR); and (8) intelligent homes. These technologies were mainly enabled by advances occurring in the fields of AI and subset technologies (eg, natural language processing [NLP], computer vision, and speech recognition), robotics, sensor technology, and advances in connectivity and computing (eg, fifth-generation cellular wireless [5G] and edge computing). The following sections provide a summary of each of these emerging technologies.

#### Assistive Autonomous Robots

Assistive robotics is a field within robotics concerned with developing robots that can assist people to manage their physical and social difficulties [54]. Assistive robotics was identified in some documents as one of the main emerging robotic developments with potential care and support applications [55-58]. This increased interest could be largely attributed to advances in AI, sensors, and human-computer interfaces [56-58]. For example, robotic dexterity, or the ability to grasp or manipulate objects, has recently experienced significant development owing to improvements in AI systems’ ability to learn via trial and error [56]. If these learning abilities are significantly improved in the future, robotic dexterity might be able to support some household and self-care tasks for older people, such as getting out of bed and assembling gadgets [56]. Wearable robotics, including suits and exoskeletons, are also expected to be used for mobility needs of older people in the near future because of reductions in the cost and size of these technologies [57,59,60]. In addition, brain-computer interfaces, one of the recent advances in human-computer interfaces, are also allowing new ways to control robotic prostheses and exoskeletons by creating neural bypasses [58,61]. These interfaces could enable potential applications for assistive robots in the rehabilitation of patients with severe paralysis, although they are still in the early stages of development [58,61]. Various ongoing projects are also capitalizing on AI and other enabling technologies to improve the autonomy of robots in real-life situations as well as to improve verbal and nonverbal interactions with end users, including older people [61]. For example, one research project is currently working on improving the image sensing and vision processing and control systems of robots, whereas another project is improving robots’ ability to analyze sounds in the environment [61]. Some of these efforts could translate into more autonomous robots in the future that are adaptive to older people’s environment and needs.

#### Self-Driving Vehicles

Self-driving vehicles were described in the reviewed documents as an emerging technology that could create new models of transportation, improve road safety, and reduce traffic congestion [57-59,62-65]. This technology could, therefore, potentially contribute to some of the transportation needs of older people in the future. However, some of the potential benefits of self-driving vehicles will depend largely on the level of automation that can be achieved. To date, significant progress has been made in creating semiautonomous vehicles (eg, Tesla autopilots), where the vehicle performs some automated functions; however, the driver’s engagement is still necessary [57,63]. A significant amount of work is also ongoing to achieve higher levels of automation [57,62-64], with some progress made recently in vehicles’ capabilities to perform all driving tasks in predefined geolocations (eg, Waymo’s autonomous taxis) [62]. However, developing fully autonomous vehicles, where they can perform all driving tasks in any environment, is complex and might be difficult to achieve in the near future [57].

#### AI-Based Health Apps and Wearables

AI and other enabling technologies are driving the development of new generation of smart apps and wearables [56,66-74]. These apps and wearables can potentially support older people to meet their needs in psychological support, self-care, and access to health care domains. For example, AI-based chatbots are at the core of a new wave of smart apps designed to provide advice to support treatments of chronic conditions, such as cognitive behavioral therapy for mental health [56,63,70]. Some of these AI chatbots also offer medical triage and advice about possible disease diagnosis before seeing doctors (eg, the Babylon app and Ada Health Companion) [69,70]. In addition, wearables such as smart watches and textiles are moving from only tracking fitness and physical activities to measuring physiological parameters and vital signs such as heart rate, electrocardiogram, and blood oxygen [56,63,74]. These developments allow wearables linked to smart apps and AI-based systems to detect early signs of disease exacerbation and help prevent further health deterioration [56,74]. The role of this new generation of wearables in the remote care of long-term conditions, including chronic obstructive pulmonary disease and mental health, is currently being investigated in several research projects in the United Kingdom [61,75].

#### New Drug Release Mechanisms

The analysis identified emerging drug release mechanisms that could offer new ways for administering medications [76-78]. Some of these drug release mechanisms are enabled by developments in sensors, AI, and other enabling technologies. For example, digital pills have been developed to deliver drugs automatically using a system that involves biosensors, smart apps, and wearable sensors [77]. This development was identified in 2016 as a trend that could have transformative impact on health [77], although it has not been widely discussed in the subsequent years. DNA origami is another development that could have potential drug delivery applications in the future [76]. These nanolevel DNA folded structures could benefit from developments in AI and sensors to act as nanorobots that could be programmed to deliver targeted therapy [76]. However, these developments are still in the early stages.

#### Portable Diagnostics

Emerging developments of point-of-care diagnostics, particularly those using smartphones, could facilitate access to health care for older people [55,57,77,79,80]. Significant work is currently ongoing to enhance sensors’ ability to detect various metabolites in body fluids and to enable spectroscopy in diagnostic devices [79,80]. These projects are paralleled with efforts to improve the detection speed, size, cost, and accuracy of these sensors [80]. Portable diagnostics equipped with these kinds of sensors are expected to bring disease diagnosis closer to patients, for instance, in the home environment [55,57,80]. However, it was not clear from the reviewed documents whether end users of these diagnostics will include patients themselves. In addition, the need to improve the diagnostic process and link data from these devices to care services and pathways has been identified [55]. Improving the process of diagnosis is also an area where AI advances hold some promising potential [71,75].

#### Voice-Activated Devices

Voice-based interfaces are one of the main emerging user interfaces identified in the documents, enabled by advances in numerous technological fields, including AI, speech recognition, and NLP [57,81-83]. These interfaces, sometimes referred to as conversational interfaces, virtual personal assistants, chatbots, or digital helpers, use end users’ speech or voice as a means to interact with the technology [57,81-83]. Voice-based interfaces have the potential to support older people in the self-care, access to health care, and social life domains. For example, voice-activated devices, such as Google Assistant and Amazon’s Alexa, can act as home digital helpers that assist older people with tasks such as providing information, medication reminders, video calling, and home entertainment [82]. These devices can also be used as platforms to control various home appliances and contribute to creating automated home experiences [82]. Significant work is also ongoing to enable voice-based interfaces to assist with more complex tasks such as web-based medical triage and self-management of chronic conditions [58,69,71,73].

#### Virtual, Augmented, and Mixed Reality

VR, AR, and MR are other emerging user interfaces identified from the analyzed documents [64,73,81,83-88]. These interfaces use a virtual world (VR) or a combination of virtual and real worlds (AR or MR) to enable immersive digital experiences [57,59,84-86]. VR, AR, and MR have the potential to support older people in social life, psychological health, and domestic life domains. For example, these emerging user interfaces are expected to enable more immersive experiences in various aspects of everyday life, including web-based home shopping, leisure activities, and communication, through the use of devices such as headsets, smart glasses, and new generations of smartphones [83,87,88]. An increased interest is also observed recently on the potential of VR, AR, and MR to support the management of mental health conditions [55,83]. Emerging advances in connectivity (eg, 5G mobile network) are also expected to improve users’ experience with VR, AR, and MR interfaces by enabling visual data transfer and processing without lags [81,88].

#### Intelligent Homes

Intelligent homes that are adaptive to users’ needs and preferences is an emerging technology that could have potential care and support applications for older people [61,82,85,87-90]. Intelligent homes are largely enabled by the internet of things (IoT) technology—a system that transfers and processes data from a group of internet-connected physical devices [63,91]. For example, IoT home systems enable automated home experiences by allowing home devices, such as lights, heat, voice-activated devices, and even mobile robots, to connect and exchange information with each other [76,82,85,91,92]. IoT systems could also be used for remote health monitoring in the home environment through monitoring and detecting changes in health and activity patterns [54,63,90,91]. Home automation experience is also expected to improve in the near future owing to advances seen in network connectivity and computing paradigms (eg, 5G and edge computing) [81,89]. In addition, artificial emotional intelligence, an emerging field within AI concerned with detecting emotions, could potentially enable the development of intelligent home devices that can adapt to users’ verbal and nonverbal behaviors [84].

## Discussion

### Principal Findings

The aim of this review was to gain an overview of emerging technologies with potential care and support applications for older people. The analysis identified 8 emerging technologies that could potentially be used to meet older people’s care needs in self-care, domestic life, mobility, psychological support, social life, and access to health care. These emerging technologies were assistive autonomous robots; self-driving vehicles; AI-based health apps and wearables; new drug delivery systems; portable diagnostics; voice-activated devices; intelligent homes; and VR, AR, and MR. Some of these technologies are recognized in the empirical literature as emerging developments that could have care applications for older people. For example, Sapci and Sapci [41] identified smart homes as an innovative assistive technology that could support aging in place. VR, self-driving vehicles, and IoT-enabled home devices were also identified in a more recent study as emerging technologies that could support older people manage health and maintain their independence [93]. Similarly, increased interest has been observed in the literature in recent years to explore the potential of assistive robots, smart homes, and voice-activated devices to support the care of older people [94-100]. It is also worth noting that many of the care applications of the emerging technologies identified in this review were health related. This might be because of the interests of the organizations included in this review. However, it could also mean that some ambiguity is still associated with potential uses of these emerging technologies in other care domains. Ambiguity regarding potential applications is indeed one of the main characteristics of emerging technologies [28] and could have influenced the applications presented in this review.

This review also highlighted that emerging technologies are at different levels of development. Some, for instance, are at early phases of development such as DNA origami, whereas others are being trialed for care applications such as using AI chatbots and VR for mental health management. However, despite many documents discussing the potential of these technologies to support various care and support domains, very few have mentioned the inclusion of older people in the design of these technologies. The needs and functional preferences of older people can indeed be overlooked during the development and design of technology [36,101]. This could result in the development of technology products that do not meet the care needs of older people, hindering their adoption by this population [93]. Using human-centered design principles and involving older people during the different stages of technology design and development will therefore be important to develop products that are desirable and usable by older people [25,101,102]. Emerging technologies identified in this review, in particular, offer an exceptional opportunity to achieve this, given that many are still in the early phases of development. In addition, it will be important to involve other stakeholders in the design process, such as family carers and care professionals, to ensure that the developed products are supported by older people’s formal and informal care systems [102].

This review also highlighted the complexity of recent technological developments, requiring a distinction to be made during the analysis phase between enabling technologies and those that could potentially be used to meet care needs of older people. Recent waves of technological developments are well recognized for their interdependencies, where new innovations are often the outcome of interactions between various fields [103,104]. Self-driving vehicles and intelligent homes, identified in this review, are good examples of innovations resulting from interactions between various technological fields, including sensors, AI, robotics, and advanced network connectivity.

These complex interactions will need to be taken into account when developing technology products targeting older people, as this could mean the need to draw on knowledge from various technological fields.

This review has several strengths. One of the strengths is following a systematic approach to search the gray literature. This systematic approach might have overcome some of the challenges associated with searching gray literature and reduced the possibility of missing key documents. Another strength is disentangling some of the complexities associated with recent technological developments to provide an overview of emerging technologies with potential care and support applications for older people. In addition, the inclusion of technological developments that were described only with the key attributes of emerging technologies may have helped overcome some of the inconsistencies associated with defining the term in the reviewed documents.

Finally, this review was exploratory in nature, where it aimed to identify emerging technological developments that could potentially be used to meet the care and support needs of older people. Therefore, issues around technology acceptability, feasibility, adoption, and ethical considerations were beyond the scope of this review. However, the results of this review will inform future work that will explore some of these issues and investigate which of the technologies identified in this review has most potential to meet the care and support needs of older people. It will involve working closely with a panel of technology experts to prioritize these technologies. This review also resulted in some implications for future research. It reinforced the importance of co-designing technology solutions and involving older people and other stakeholders, such as carers and care professionals, at various stages of technology design and development. In addition, research and development related to emerging technologies might need to be interdisciplinary, given the interdependencies and complexity of recent technological advances.

### Limitations

There are some limitations that need to be acknowledged. The search and analysis processes were conducted primarily by the first author (SA). There is a possibility that this was influenced by the author’s own perceptions and interpretations. However, the process of the search and analysis was discussed regularly with the research team to minimize potential bias. There is also a possibility that the search strategies missed key literature in other languages. In addition, many of the included documents were published in the United Kingdom and the United States; therefore, there is a possibility that this review missed some technological developments occurring in other parts of the world. Finally, there might be a need to consolidate the review findings with experts’ consultations or studies from peer-reviewed literature, as some gray literature sources do not go through a rigorous review process.

### Conclusions

In summary, this review provided an overview of emerging technologies with potential care and support applications for older people. A total of 8 emerging technologies were identified, including self-driving vehicles, assistive autonomous robots, intelligent homes, VR and AR, AI-enabled apps and wearables, voice-activated devices, portable diagnostics, and new drug release mechanisms. These technologies were at different levels of development, with some being trialed for care applications, whereas others are in the early stages of development. The results of this review can be used by researchers, designers, and developers to gain an overview of the topic investigated as well as co-design applications of some of the technologies identified with older people. Formal and informal carers might also be interested in exploring some of the technologies identified to meet the care needs of their care recipients. The findings of this review will be used by the research team to investigate which of the emerging technologies identified has the most potential to meet the care and support needs of older people.

## Appendix

Multimedia Appendix 1 Characteristics of the documents included in the final analysis.

## Abbreviations

5G: fifth-generation cellular wireless
ADL: activity of daily living
AI: artificial intelligence
AR: augmented reality
EPSRC: Engineering and Physical Sciences Research Council
IoT: internet of things
MR: mixed reality
NLP: natural language processing
PRISMA: Preferred Reporting Items for Systematic Reviews and Meta-Analyses
VR: virtual reality
