# Supplementary material for: Emerging Technologies With Potential Care and Support Applications for Older People: Review of Gray Literature
Source: JMIR Aging. 2020 Aug 11;3(2):e17286. doi: 10.2196/17286 (PMC7448188; doi:10.2196/17286)
Supplement: Multimedia Appendix 1 [file aging_v3i2e17286_app1.docx]

**Multimedia appendix 1.** Characteristics of the documents included in the final analysis

| **No.** | **Title of the document** | **Publishing body** | **Year of publication** | **Country** | **Type of grey literature document** | **Purpose of the document** | **Type of technology discussed** | **Potential care and support applications** |
| --- | --- | --- | --- | --- | --- | --- | --- | --- |
| 54 | Top 10 Emerging Technologies 2019 | World Economic Forum | 2019 | International | Report | Review technological breakthroughs that could have an impact on society and economy. | - Social robots - Collaborative telepresence (virtual physical presence) through the use of VR and AR | Social robots:   - Reduce stress for older people with Alzheimer’s disease - Act as a wellness aide, reminding older people to take walks and medications and to call family members   Collaborative telepresence through the use of VR/AR   - Socialising - Patient remote monitoring/counselling |
| 55 | The Topol review, preparing the healthcare workforce to deliver the digital future, an independent report on behalf of the secretary of state for health and social care | NHS | 2019 | UK | Report | Review technological developments (AI, robotics, genomics, digital medicine) that could have an impact on the healthcare workforce | - Smartphone apps - Sensors and wearables - Voice assistants - Virtual and augmented reality - Intervention and rehabilitative robotics - Convergence of AI, sensors and genomics | Smart apps   - Self-management of chronic conditions   Sensors and wearables   - Portable diagnostics - Remote monitoring - Self-management of chronic conditions   Voice assistants   - Find information - Control home automation devices - Manage entertainment systems - Perform administrative tasks   VR/AR   - Mental health support such as in anxiety, post traumatic syndrome - Pain management   Robotics   - Improve functionality of patients with physical disability. - Social or companion robots   Convergence of AI, genomics and sensors   - Virtual medical coaches that could assist with self-managing conditions and real-time remote monitoring |
| 56 | MIT Technology Review Volume 122 Issue 2 | MIT | Mar 2019 | US | Review articles | Review 10 big technological breakthroughs could have potential impact on society, medicine, and economy. | - Robotic dexterity - Wearables - Driverless cars | Robotic dexterity   - Could potentially assemble gadgets, load dishwashers and help elderly out of bed   Wearables   - Monitor ECG   Driverless cars   - New transportation model |
| 57 | World Economic and Social Survey- frontier technologies for sustainable development | UN | 2018 | International | Report | Investigates frontier technologies and how can they can be harnessed to achieve sustainable development goals (including the 3^rd^ goal-ensure healthy lives and promote well-being for all at all ages) | - Conversational AI systems - Smartphone-based portable diagnostics - Assistive robots - Autonomous vehicles | Conversational AI   - Psychological support for patients with depression or anxiety.   Smartphone-based portable diagnostics   - Substitutes expensive medical devices   Assistive robots   - Support those who are paralyze or disable to walk   Autonomous vehicles   - New transportation models that could reduce accident rates |
| 58 | MIT Technology Review Volume 120 Issue 2. | MIT | Mar 2017 | US | Review articles | Review 10 big technological breakthroughs could have potential impact on society, medicine, and economy. | - Robotic prothesis controlled by brain-computer interfaces - Self-driving vehicles - Robotic grocery store - Home robots | Robotic prothesis   - Moving limbs through thoughts - Support the disabled to walk   Robotic grocery store   - Food shopping   Home robots   - Offer companionship - Play music or control their smart devices from anywhere in the home - Surveillance   Self-driving vehicles   - New transportation model |
| 59 | Project breakthrough- disruptive technology executive briefs. | PA consulting | 2017 | International | Executive briefs | Review technological breakthroughs and how can they can be used to achieve sustainable development goals (including the 3^rd^ goal-ensure healthy lives and promote well-being for all at all ages) | - AI systems (e.g. AI chatbots) - Internet of things (IoT) - Virtual reality - Autonomous vehicles - Autonomous robotics | AI systems   - Medical online triage   IoT   - Real-time remote monitoring by healthcare professionals - Provides individuals immediate access to information that highlights impact of behaviour on health, through the use of wearables   Virtual reality   - Support mental health treatments such as PTSD, anxiety, phobias   Autonomous vehicles   - New transportation models that could result in new ownership models   Autonomous robotics   - Exoskeletons that could enable people paralysed to walk again |
| 60 | Pionner-The science behind the theory of everything | EPSRC | 2015 | UK | Experts Magazine | Showcases EPSRC key funded research projects | - Robotic prosthetic hand controlled by the nervous system, - Robotic clothing, - Biosensors | Robotic hand   - Improves people’s lives and allow greater independence   Robotic clothing   - Help people with walking   Biosensors   - Monitor how patients use equipment or exercise during rehabilitation |
| 61 | Information and communication technologies EPSRC research theme. | EPSRC | 2019 | UK | List and description of ongoing research projects | Describes EPSRC’s current portfolio of research grants in the information and communication technologies theme. | - Wearable wireless technology (wearable + mobile app) - Neural interfaces or brain machine interfaces (BMIs) - Socially assistive robots | Wearable wireless technology   - Early detection of potential exacerbations in COPD which could help in minimising likelihood of progression - Self-management of multi-morbidities - Remote monitoring and early intervention   Neural interfaces   - Could potentially help an amputee to control a robotic prosthetic - Could support paraplegic to control a mobility aid   Socially assistive robots   - Improve robots’ ability to learn and adapt to acoustic environment, which could result in better response to users’ environment |
| 62 | MIT Technology Review Volume 121 Issue 4 | MIT | Jul 2018 | US | Review articles | Review and provides updates about recent technological advances, focusing on the human and social dimensions of the technology | - Self- driving vehicles - AI-enabled robots | Self-driving vehicles   - New transportation models that could result in ownerless cars.   AI-enabled robots   - Autonomous robotic arms that could assemble and process packages |
| 63 | MIT Technology Review Volume 119 Issue 5 | MIT | Sep 2016 | US | Review articles | Review and provides updates about recent technological advances, focusing on the human and social dimensions of the technology | - Self-driving vehicles | - New transportation model |
| 64 | MIT Technology Review Volume 119 Issue 6. | MIT | Nov 2016 | US | Review articles | Review and provides updates about recent technological advances, focusing on the human and social dimensions of the technology | - Autonomous vehicles - Virtual reality | Autonomous vehicles   - New transportation models   Virtual reality   - Leisure activities such as gaming, and virtual travel |
| 65 | Deep shift 21 ways software will transform global. | World Economic Forum. 2015. | 2015 | International | Report | Review technological breakthroughs that could have an impact on society and economy | - Driverless cars - The connected home - The internet of things - Wearables - Implantable technologies - Vision interfaces | Driverless cars   - New transportation models   The connected home   - Support independent living for older people - Connected robots can help with domestic tasks such as vacuum cleaning   The internet of things   - Improve quality of life   Wearables   - Self-management of conditions   Implantables   - Monitoring health parameters - Support communicating thoughts by reading brainwaves   Vision interfaces   - Support people with disability to manage interactions and movement through speaking, typing, moving and immersive experience |
| 66 | Ethical, social, and political challenges of artificial intelligence in health | The Wellcome Trust | 2018 | UK | Report | Examine how AI is being used in healthcare, and how it could be used in the near future. | - Patient facing applications such as chatbots and virtual assistants - Assistive robots | Patient facing applications   - Answer condition specific questions   Assistive robots   - Embedded systems connected to mobile assistive robots to assist with smart home experience - Take over physical tasks such as lifting patients from bed - Remind patients to take medications |
| 67 | MIT Technology Review Volume 121 Issue 6. | MIT | Nov 2018 | US | Review articles | Review and provides updates about recent technological advances, focusing on the human and social dimensions of the technology | - AI-enabled apps | AI-enabled apps   - Virtual medical coaches that use physiological, genetic , environmental, behavioural parameters collected by the app - Medical triage (e.g. GP at hand) - Measuring cognition and emotional health and share it with patients and healthcare providers |
| 68 | Nuffield Council on Bioethics. Artificial Intelligence in Healthcare and Research | Nuffield | 2019 | UK | Brief report | Examines the current and potential healthcare applications of AI | - Patient facing AI-enabled apps (e.g. virtual assistants and chatbots) | - Offer personalised health assessments and home care advice - Provide information regarding symptoms - Assist with the self-management of chronic conditions - Assist with the monitoring of medication adherence - Early detection of health deterioration and early intervention and avoid hospital admission |
| 69 | Confronting Dr Bot, creating a people powered future for AI in health. | NESTA | 2018 | UK | Report | Explore how AI might be used in UK health system, and how AI-enabled healthcare might look and feel from the citizen point of view. | - AI-enabled apps (e.g. chatbots) | - Advice and triage before seeing a doctor - Remote monitoring, early detection of health deterioration - Support mental health treatment (CBT) |
| 70 | The promise of health tech | Public | 2018 | UK | Report | Review of how health start-ups and digital innovators are transforming the NHS | - Apps, portable diagnostics, smart drug delivery | - Support self-care and integrated health and care management |
| 71 | Thinking on its own- AI in the NHS. | Reform | 2018 | UK | Report | Review areas where artificial intelligence (AI) could help the NHS become more efficient and deliver better outcomes for patients. | - AI-enabled wearables - AI-enabled apps | AI-enabled wearables   - Monitor information and vital systems such as heart rate and AI systems can interpret information and facilitate better knowledge access by patients   AI-enabled apps   - Self-management of chronic conditions such as diabetes |
| 72 | MIT Technology Review Volume 118 Issue 4. | MIT | Jul 2015 | US | Review articles | Review and provides updates about recent technological advances, focusing on the human and social dimensions of the technology | - Medical wearables - Text-to-speech interface | Wearables   - Monitoring common chronic illnesses   Text-to-speech interface   - Uses audio or tactile feedback to help users scan their fingers along lines of text |
| 73 | Top 10 Emerging Technologies 2018. | World Economic Forum | 2018 | International | Report | Review technological breakthroughs that could have an impact on society and economy. | - Virtual and augmented reality - AI-enabled conversational agents - Implantable drug making cells | VR/AR:   - Leisure activities such as gaming   Chatbots:   - Digital assistant e.g. provide advice, debate, find information   Implantable drug making cells:   - new drug release mechanisms |
| 74 | The NHS at 70: what will new technology mean for the NHS and its patients? | Kings Fund | 2018 | UK | Report | This report illustrates the areas where artificial intelligence (AI) could help the NHS become more efficient and deliver better outcomes for patients. | - Smart apps and wearables - AI algorithms | Smart apps and wearables   - Remote monitoring, early detection of health deterioration and intervention - Self-management of chronic conditions   AI algorithms   - Online medical triage |
| 75 | EPSRC healthcare technologies research theme. | EPSRC | 2019 | UK | List and description of ongoing research projects | Describes EPSRC’s current portfolio of research grants in the healthcare technologies research theme | - Healthcare sensors - Wellness sensors + AI + mobile app - AI-enabled wheelchair - Soft wearable rehabilitative devices - New hearing aids | Healthcare sensors   - Support self-management of chronic conditions through using AI algorithms that analyse information from healthcare home sensors with information from GP and hospital visits.   Wellness sensors + AI + mobile app   - Help patients follow treatment guidelines for multiple morbidities and will personalise treatment advice based on details collected on patients.   AI-enabled wheelchair   - Support people with disability to drive powered wheelchair and give them an opportunity for more independent mobility.   Soft wearable rehabilitative devices   - Support people to walk, stand and to move from sitting to standing with the support of physiotherapist or in the absence of a therapist   New hearing aids   - Improve how hearing devices deal with speech in noise which could improve many aspects of health and well-being for older people (e.g. social situations) |
| 76 | Health and Healthcare in the Fourth Industrial Revolution Global Future Council on the Future of Health and Healthcare | World Economic Forum | 2019 | International | Report | Provide an insight on the impact of the fourth industrial revolution on health and healthcare in the future. | -Internet of medical things (IoMT)  -Nanotechnology  -Robots  - Virtual and augmented reality (VR, AR) | Internet of medical things (IoMT)   - Remote monitoring - Virtual home assistants that could support older people with taking medications, coordinating care and getting in touch with family. - Medical adherence tracking through smart pill system - Emergency assistance and ensuring safety at home (e.g. for patients with dementia)   Nanotechnology   - DNA origami that could have drug delivery applications   Robots   - Support elderly care   VR, AR   - Support treatment of mental health conditions such as post-traumatic stress disorder and phobias. |
| 77 | The digital revolution: eight technologies that will change health and care. | King’s Funds | 2016 | UK | Extended article | Examine the technologies most likely to change health and care over the next few years | - Smart phone apps - Portable diagnostics - Smart assistive devices - Smart or implantable drug delivery mechanisms | Smart phone apps   - Deliver CBT   Portable diagnostics   - Hospital-level diagnosis at home   Smart assistive devices   - Support people with long-term conditions and people with disability to perform tasks and activities such as tremor spoon   Smart or implantable drug delivery mechanisms   - Automating drug release in the body |
| 78 | EPSRC engineering research theme. | EPSRC | 2019 | UK | List and description of ongoing research projects | Describes EPSRC’s current portfolio of research grants in the engineering theme. | - New drug delivery system | New drug delivery system   - Provides long-acting drug release |
| 79 | Pionner- Home improvements- Science and engineering for a hi-tech low carbon world | EPSRC | 2018 | UK | Experts Magazine | Showcases EPSRC key funded research projects | - Low cost health sensor (that can measure metabolites such as lactate, glucose) - Smart textiles | Low cost health sensor   - Allow cheap, quick and accurate patient health conditions   Smart textiles   - Ease pain through delivering a small electrical current to interfere with pain signals and stimulate the body to release of natural endorphins |
| 80 | EPSRC Sensors and instrumentation research area. | EPSRC | 2019 | UK | List and description of ongoing research projects | Describes EPSRC’s current portfolio of research grants in the sensors  and instrumentation theme. | - Advanced e-textiles for wearable therapeutics - Wearable sensors - Acoustic signal processing and scene analysis for socially assistive robots | E-textiles   - Health monitoring (e.g. ECG) and electroencephalography (EEG) and treatment (e.g. pain relief and rehabilitation)   Wearable sensors   - Vital signs monitoring and continuous remote monitoring   Socially assistive robots   - Improve robots’ ability to learn and adapt to acoustic environment, which could result in better response to users’ environment |
| 81 | Tech Trends 2019 Beyond the digital frontier | Deloitte. | 2019 | International | Report | Examines emerging technologies that are likely to disrupt businesses over the next 18 to 24 months. | - Intelligent interfaces (e.g. conversational interfaces, virtual reality, brain-controlled interfaces, gait analysis, bioacoustics sensing, emotion detection, muscle-computer interfaces) | Intelligent interfaces   - Personalised shopping experience, as companies are expected to use intelligent interfaces to track customers habits |
| 82 | MIT Technology Review Volume 120 Issue 5. | MIT | Sep 2017 | US | Review articles | Review and provides updates about recent technological advances, focusing on the human and social dimensions of the technology | - Voice-enabled assistants | - Leisure activities (e.g. listen to audiobooks) - Keep in touch with family news - Control home appliances (home automation) - Home entertainment - Shopping - Potential to support those who cannot use telephone, mobile or with limited mobility |
| 83 | MIT Technology Review Volume 119 Issue 2 | MIT | Mar 2016 | US | Review articles | Review 10 big technological breakthroughs could have potential impact on society, medicine, and economy. | - Smart homes - Virtual reality bike - Conversational interfaces | Smart homes   - Automating home experience by connecting products   Virtual reality bike   - Immersive experience for exercising indoor   Conversational interfaces   - Easier use of smartphones - Easier to talk to home appliances and robots |
| 84 | MIT Technology Review Volume 120 Issue 6. | MIT | Nov 2017 | US | Review articles | Review and provides updates about recent technological advances, focusing on the human and social dimensions of the technology | - Artificial emotional intelligence | Artificial emotional intelligence   - Monitor drivers for fatigue, distraction and frustration resulting in personalising car driving experience - Track emotions and early detection of mental related disorders - Facilitate communication with conversational interfaces |
| 85 | MIT Technology Review. 2016. MIT Technology Review Volume 119 Issue 4. | MIT | Jul 2016 | US | Review articles | Review and provides updates about recent technological advances, focusing on the human and social dimensions of the technology | - Robotic home | Robotic home   - Home automation that include connecting home appliances, entertainment systems, heating, air conditioning |
| 86 | MIT Technology Review Volume 119 Issue 1 | MIT | Jan 2016 | US | Review articles | Review and provides updates about recent technological advances, focusing on the human and social dimensions of the technology | - Virtual reality | - Enables immersive experience for communication and entertainment |
| 87 | MIT Technology Review. 2017. MIT Technology Review Volume  120 Issue 4 | MIT | Jul 2017 | US | Review articles | Review and provides updates about recent technological advances, focusing on the human and social dimensions of the technology | - Virtual reality - Smart home assistant - Self-driving vehicles | Virtual reality   - Socialising - Communication   Smart home assistant   - Monitor home and ensure home safety   Self-driving vehicles   - New transportation model |
| 88 | Tech Trends 2018 The symphonic enterprise | Deloitte. | 2018 | US | Report | Examines emerging technologies that are likely to disrupt businesses over the next 18 to 24 months. | - Digital reality (virtual, augmented, mixed reality) | Digital reality   - Video conferencing - Online shopping - Leisure activities (e.g. gaming)   AI systems (e.g. IBM Watson) |
| 89 | MIT Technology Review Volume 119 Issue 3 | MIT | May 2016 | US | Review articles | Review and provides updates about recent technological advances, focusing on the human and social dimensions of the technology | - AI systems (e.g. IB Watson) | - Could potentially advice people on diet and fitness |
| 90 | Technological innovations for health and wealth for an ageing global population | World Economic Forum. | 2016 | International | White paper | Evaluate technological and social innovations for healthy ageing with applicability to the financial services industry | - Robotic assistants and prosthetic devices - Telehealth - Sensory technologies - In-home sensing, wearables, remote patient monitoring - Cognitive health technologies such as brain training applications - Virtual communities, real-time videoconferencing, interactive games, social support network or blogging platform | Robotic assistants and prosthetic devices   - Detect and prevent falls   Telehealth   - Improve access to healthcare services   Sensory technologies   - Support hearing, vision and tactile functions   Social media, virtual communities etc.   - Support social connectivity and emotional health with carers as mediators between older people and technology   In-home sensing, wearables, remote patient monitoring   - Screen for wandering and falls, - Monitor vital signs - Ensure consumption and refills of prescribed medication   Cognitive health technologies such as brain training applications   - Enhance cognition by improving memory, sustaining attention and facilitating dual-task performative - Leverage advances in AI and cognitive science to support independence among older people |
| 91 | Top 10 Emerging Technologies 2016. | World Economic Forum. | 2016 | International | Report | Review technological breakthroughs that could have an impact on society and economy | - Internet of things (IoT) /Internet of nanothings - Autonomous vehicles | IoT   - Provide detailed picture of the home   Autonomous vehicles   - New transportation models |
| 92 | Top 10 Emerging Technologies 2015. | World Economic Forum. | 2015 | International | Report | Review technological breakthroughs that could have an impact on society and economy | - Robotics | Robotics   - Support older people out of bed - Support rehabilitation (e.g. stroke victims in regaining control of their limbs) |
